
JOURNAL INFORMATION
==============================
NLM Title Abbreviation: Wirtschaftsdienst
Journal ID: Wirtschaftsdienst
NLM Journal ID: 101086612

Wirtschaftsdienst (Hamburg, Germany : 1949)

ISSN: 0043-6275

ARTICLE INFORMATION
==============================
PMCID: PMC7237623
PMID: 32454538
DOI: 10.1007/s10273-020-2638-y
Article ID: 2638
Article version: 1
Subjects: Leitartikel

Wissenschaft braucht Daten

Science needs data

Riphahn Regina T. regina.riphahn@fau.de

grid.5330.5 0000 0001 2107 3311 LS Statistik und emp. Wirtschaftsforsch., Friedrich-Alexander Universität, Lange Gasse 20, 90403 Nürnberg, Deutschland
Electronic publication date: 2020 May 20
Print publication date: 2020
Volume: 100
Issue: 5
First page: 306
Last page: 307
Copyright: © Der/die Autor(en) 2020
License: Open Access Dieser Artikel wird unter der Creative Commons Namensnennung 4.0 International Lizenz veröffentlicht, welche die Nutzung, Vervielfältigung, Bearbeitung, Verbreitung und Wiedergabe in jeglichem Medium und Format erlaubt, sofern Sie den/die ursprünglichen Autor(en) und die Quelle ordnungsgemäß nennen, einen Link zur Creative Commons Lizenz beifügen und angeben, ob Änderungen vorgenommen wurden.
Die in diesem Artikel enthaltenen Bilder und sonstiges Drittmaterial unterliegen ebenfalls der genannten Creative Commons Lizenz, sofern sich aus der Abbildungslegende nichts anderes ergibt. Sofern das betreffende Material nicht unter der genannten Creative Commons Lizenz steht und die betreffende Handlung nicht nach gesetzlichen Vorschriften erlaubt ist, ist für die oben aufgeführten Weiterverwendungen des Materials die Einwilligung des jeweiligen Rechteinhabers einzuholen.
Weitere Details zur Lizenz entnehmen Sie bitte der Lizenzinformation auf http://creativecommons.org/licenses/by/4.0/deed.de.
License URL: https://creativecommons.org/licenses/by/4.0/

issue-copyright-statement: © The Author(s) 2020

==============================
Regina T. Riphahn ist Inhaberin des Lehrstuhls für Statistik und empirische Wirtschaftsforschung an der Friedrich-Alexander-Universität Erlangen-Nürnberg und Vorsitzende des RatSWD.

Unter dem Leitmotiv „Gesellschaft braucht Wissenschaft – Wissenschaft braucht Daten“ stand 2020 die 8. Konferenz für Sozial- und Wirtschaftsdaten des Rates für Sozial- und Wirtschaftsdaten (RatSWD). Als wir das Thema festlegten, war nicht absehbar, dass die Verfügbarkeit von Daten einmal so sehr in der öffentlichen Wahrnehmung stehen würde, wie das heute der Fall ist. Die Corona-Pandemie bringt Daten in die Schlagzeilen: Jeden Tag wird der neueste Stand zu Neuinfektionen, Genesenen, Verdopplungszeit und Reproduktionswert durchgegeben und von der Bevölkerung aufmerksam verfolgt. In den Abendnachrichten wurden Statistiker gebeten, einem Millionenpublikum die Bedeutung von Zufallsstichproben zu erklären (Tagesschau vom 28.4.2020).

Schaut man einmal hinter die Kulissen und fragt nach der Verlässlichkeit der Maße, mit denen wir die tägliche Corona-Lage einschätzen, so beginnen die vormals so eindeutigen Fakten zu verschwimmen. Wie sollen die Todesfälle gezählt und abgegrenzt werden, „mit“ oder „durch“ COVID-19? Was bedeutet der Zusammenhang zwischen der Zahl der Neuinfektionen und der Testhäufigkeit? Wie aktuell sind die Daten, die hinter der Reproduktionszahl stecken und wie wichtig sind Modellannahmen? Die Relevanz verlässlicher Daten wird spürbar, wenn man mit den Zahlen arbeiten muss, die man hat. Diese Situation ist der empirischen Wirtschafts- und Sozialforschung nicht unbekannt, so geht es uns in vielen Feldern. Schwerer zu verstehen ist, dass wir oft mit beschränkt aussagekräftigen Daten arbeiten müssen, obwohl es gute Daten gibt, die aus öffentlichen Mitteln finanziert wurden. Sie werden der empirischen Forschung vorenthalten.

Vier Beispiele: (a) Vor Corona hat uns der Wohnungsmarkt beschäftigt. Viele wichtige Fragen ließen sich mit Transaktionsdaten zu den Verkäufen von Häusern und Grundstücken beantworten. Diese Daten liegen den Gutachterausschüssen vor. In manchen Regionen werden sie der Forschung zur Verfügung gestellt, in anderen nicht – gerne wegen des „Datenschutzes“. In anderen Ländern ist das anders. In Großbritannien ist jede Transaktion einer Wohneinheit mit Verkaufspreis, Wohnungstyp, Adresse und Transaktionsdatum über eine staatliche Stelle frei verfügbar. Das ermöglicht Forschung und unterstützt damit evidenzbasierte Politikgestaltung. (b) Die Kultusministerkonferenz hat 2003 beschlossen, Schülerkerndatensätze zu erstellen, die Bildungsbiografien in Deutschland abbilden. Auch aus den Daten von Schuleingangsuntersuchungen könnten wir viel lernen, etwa über die Wirksamkeit von Reformen und die Relevanz von Rahmenbedingungen. Allein, die Länder stellen diese Daten in der Regel nicht zur Verfügung und bereiten sie teilweise gar nicht erst auf. (c) Viele finanzwissenschaftliche Analysen etwa zu Reformwirkungen, zu Vollzugsdefiziten im Steuersystem oder zu Verteilungseffekten können nicht erfolgen, da Steuerdaten nicht verfügbar sind. Vieles wird gar nicht erst erhoben. Aber vielfach werden selbst vorliegende Daten der Landesfinanzbehörden für die Forschung nicht bereitgestellt. (d) In vielen Bereichen staatlichen Handelns fehlt der klar geregelte Zugang der Forschung zu einschlägigen Registern. Dies gilt insbesondere für Melderegister, Ausländerzentralregister, Bildungsregister, und Mortalitätsregister. Hier würde eine Registermodernisierung – etwa ein Gesetz, das den grundsätzlichen Zugang der Wissenschaft zu Registerdaten regelt, wie es z. B. in Österreich verabschiedet wurde – weiterhelfen.

Die Datenstrategie der Bundesregierung könnte solche Problemlagen systematisch angehen und vorhandene Daten zugänglich machen. Für die genannten Fälle liegt die Lösung auf der Hand: Über den Weg der akkreditierten Forschungsdatenzentren (FDZ) können staatliche Akteure schon heute sensible Mikrodaten unter strenger Einhaltung des Datenschutzes für die Forschung bereitstellen. Die statistischen Ämter leisten das schon lange und sehr erfolgreich.

Neben dem Datenzugang tun sich Probleme in mindestens vier weiteren Gebieten auf: (a) Oft behindert der gesetzliche Rahmen zu Datenverknüpfungen innovative Forschung. Das Bundesstatistikgesetz erlaubt beispielsweise nicht das Zusammenspielen von Unternehmensdaten des Statistischen Bundesamtes mit Mikrodaten anderer Datenproduzenten oder von Surveydaten mit Registerdaten. Eine Rechtsgrundlage für Datenverknüpfungen zu wissenschaftlichen Zwecken fehlt. (b) Ein weiteres Desiderat betrifft die nutzungsfreundliche Umsetzung eines Zugangs zu Daten der amtlichen Statistik etwa vom individuellen Forschungs-Arbeitsplatz aus – in anderen Ländern problemlos möglich und gerade in Pandemiezeiten wichtig. In Deutschland erfordert ein solches Remote-Desktop-Verfahren für den Zugang zu formal anonymisierten Einzeldaten eine Anpassung von § 16 Abs. 6 Bundesstatistikgesetz. (c) Uns fehlt die gesetzlich geregelte Institution eines Datentreuhänders, der die Kooperation zwischen Forschung und Unternehmen unterstützen würde. Vertrauensvolle Zusammenarbeit mit privatwirtschaftlichen Datenanbietenden wäre für Forschungszwecke möglich, wenn für alle Beteiligten Rechte und Beschränkungen verlässlich geregelt wären. Unternehmen könnten in gesichertem Rahmen ihre Daten an einen Datentreuhänder abgeben, der Vertraulichkeit sichert und Daten der Forschung zugänglich macht. (d) Daten fehlen in vielen wichtigen Themenfeldern. So weisen die deutschen Kriminal- und Strafrechtspflegestatistiken erhebliche Lücken auf. Gegenwärtig gibt es keine bundesgesetzliche Grundlage für eine flächendeckende und einheitliche Erfassung. Die Statistiken nutzen verschiedene Zählweisen, Aufbereitungs- und Erhebungskonzepte. Eine Harmonisierung ist überfällig. Momentan sind keine Analysen möglich, die den Verlauf zwischen Tatverdacht, Verurteilung und gegebenenfalls Freilassung oder wiederholten Straftaten nachzeichnen. Erst solche Studien ermöglichen aber eine rationale Sicherheits- und Justizpolitik.

Dafür, dass Deutschland im internationalen Vergleich im Bereich der Datenbereitstellung für die Wissenschaft rückständig ist, gibt es viele Gründe. Nicht selten spielt ein hierzulande besonders intensives Datenschutz-Misstrauen eine Rolle, das empirische Forschung durch Beschränkungen bei Datenzugang und -nutzung erschwert. Hier ist es wichtig, über Motivation und Anliegen der Wissenschaft aufzuklären, die nie über die Re-Identifizierung von Individuen ihre Ziele erreicht. Datenschutz wird von der Verwaltung oft vorgeschoben, wenn es Behörden eigentlich an Personal und Kompetenz fehlt, um Forschenden den Zugang zu vorhandenen Daten zu ermöglichen. Hier ist ein systematisches Nachsteuern und verlässliche Unterstützung erforderlich. Der RatSWD engagiert sich seit vielen Jahren für Datenbereitstellung und Datenzugang. Mit aussagekräftigen Daten gelingt verlässliche empirische Forschung. In Pandemiezeiten akzeptiert jeder, dass Infektionen nur mit Tests, also Datensammlung, beherrschbar werden. Aber der Zusammenhang zwischen Evidenz und vernünftigen Entscheidungen gilt für viele Bereiche: Gute Politik braucht gute Wissenschaft und gute Wissenschaft braucht (gute) Daten. Um hier – auch über die Corona-Krise hinaus – Fortschritte zu machen, ist der Gesetzgeber gefragt. Mit dem Digitale-Versorgung-Gesetz werden Forschungsdatenzentren für die Gesundheitsforschung gefordert, ein wichtiger Schritt. Mit Unterstützung der Datenstrategie der Bundesregierung besteht derzeit die große Chance, den Datenzugang der Wissenschaft systematisch auf breiter Basis zu stärken und bestehende Hindernisse abzubauen. Dies ebnet den Weg zu neuen Erkenntnissen im gesamtgesellschaftlichen Interesse. Wir haben manches erreicht, aber es bleibt viel zu tun.

Regina T. Riphahn
Friedrich-Alexander-Universität
Erlangen-Nürnberg/RatSWD
regina.riphahn@fau.de
